# Developing and validating of the Clinical Uncertainty Measurement Questionnaire (CUMQ) among practicing physicians and clinical residents in Iran

**DOI:** 10.1186/s12909-022-03444-1

**Published:** 2022-06-16

**Authors:** Shirin Ghanavati, Hamid Reza Baradaran, Seyed Kamran Soltani Arabshahi, Shoaleh Bigdeli

**Affiliations:** Center for Educational Research in Medical Sciences (CERMS), Department of Medical Education, School of Medicine, Iran University of Medical Sciences, Tehran, Iran

**Keywords:** Validity, Reliability, Clinical Uncertainty, Questionnaire

## Abstract

**Background:**

Despite the fact that clinicians face uncertainty in their decisions, there is no comprehensive framework to measure it in medical practices which is the knowledge gap especially for Iran. Therefore, this study aimed to evaluate the reliability and validity of a Persian questionnaire which is designed to measure different determining aspects of uncertainty from clinical physicians’ perspectives in Iran.

**Methods:**

Clinical Uncertainty Measurement Questionnaire (CUMQ) has been derived from a mixed method study since March 2019 to January 2021. To exclude raw items of the questionnaire, the literature was reviewed and in-depthinterviews were implemented with 24 residents,specialists and sub-specialists in all major clinical fields which resulted in the first theoretical uncertainty in clinical decision making framework. CUMQ content validity has been evaluated using content validity index (CVI) and content validity ratio (CVR). The structural validity of the questionnaire was assessed using confirmatory factor analysis and factor loading and t-value for each indicator of uncertainty is reported. Moreover, to analyze the research model we used the Partial Least Squares (PLS) technique using the SmartPLS software. Convergent (using Average Variance Extracted (AVEs) for each latent variable) and discriminant validity (using the criteria of Fornell and Larckerand cross loading) of the model was also evaluated. After that, the quality of the model was evaluated adjustment through predictive validity (Q^2^) and effect size (f^2^). In addition, the reliability was also assessed using Cronbach’s alpha and composite reliability.

**Results:**

The CVR and CVI ranged from 0. 80 to 1. 00 which illustrates high content validity. Out of 30 items, 24 items had acceptable factor loading and remained in the questionnaire which have been categorized as five main clinical uncertainty dimensions; general determinants, individual determinants of the physician, individual determinants of patient, dynamics of medical sciences, diagnostic and instrumental limitations. The value of composite reliability and Cronbach’s alpha for all dimensions were above the threshold value of 0. 7 and the reliability has been confirmed. As AVE values were greater than 0. 5, convergent validity is confirmed. The result of Fornell-Larcker and cross-loadings also indicated that discriminant validity is well established.

**Conclusion:**

This CUMQ is as avalid and reliable instrument and a suitable tool to measure clinical uncertainty in the Iranian Medical community. However, the reliability of this questionnaire can be studied in other languages and in other countries.

**Supplementary Information:**

The online version contains supplementary material available at 10.1186/s12909-022-03444-1.

## Background

Since the time of Hippocrates, physicians have recognized that diagnosis, treatment and outcome are subject to uncertainty [1]. Although most clinical decisions are made when a physician (with all genders or even non-binary ones), based on his knowledge, experience, and available evidence, is assured [2], the complexity of healthcare systems, tasks and patient care can develop high levels of uncertainty among healthcare workers [3].

Uncertainty is a "distinct" concept, but an unclear one [4]. Uncertainty in medical decision making has several theoretical and working definitions; “lack of familiarity with the necessary information, unavailability of relevant information, inability to assess the impact of patient or disease characteristics on outcome with one versus another treatment strategy, and poor understanding of patient preferences or priorities, among others” [5]. However, Bhise et al (2018) in a systematic review study referred to a brief but relatively comprehensive definition, which is “subjective perception of an inability to provide an accurate explanation of the patient’s health problem” [6].

An exposureof a physician to a patient to make the best rational and ethical decision creates a complex enterprise where there are more questions than answers [7]. The condition of each patient as well as his environment might be different from others and could intensify factors causing clinical uncertainty [2]. Moreover, physicians themselves can be a crucial source of uncertainty [5]. In fact, unsorted array of information, informational deficits and limited knowledge of physicians could lead to uncertain decision-making [2]. Many medical decisions involve uncertain outcomes and clinical uncertainty is almost inevitable [2, 7–10]. In other words, uncertainty in medical evidence is inherent even through clinical counseling [11, 12], which results in unwanted and undesired care delays and sometimes patient harm, and more cost of care [10, 12, 13]. Nevertheless, there is no comprehensive framework to measure diagnostic uncertainty in medical practice [6]. The German dealing uncertainty questionnaire (DUQ) has been developed to measure the level of action and active reasoning in dealing with uncertainty among general practitioners (GPs). It resulted in two scales as “GP action scale” and “GP diagnostic reasoning scale”. Cronbach’s alpha for ‘GP action scale’ was 0. 75 and for ‘GP diagnostic reasoning scale’ 0. 62 [14]. Part of a recent study also applied 6 items measuring job-related situation associated with uncertainty among physicians and the reported Cronbach’s alpha was 0. 65 [15].

As many and sometimes irreparable consequences occur by uncertainty in clinical decision-making which may lead to an inappropriate patients’ management [10, 13], which Iran is not an exception, its measurement would be considered vital. However, previous questionnaires have focused on physicians’ reactions towards uncertainty [14, 16, 17]; its various dimensions and determinants among clinical physicians have received no attention in the Iranian contexts. Thus, this research has looked into different aspects of validation, such as internal consistency reliability, convergent validity, and discriminant validity for each individual item of the instruments with the second-generation statistical analysis, PLS-SEM^1^ approach. Therefore, this study aimed to evaluate the reliability and validity of the questionnaire which is designed to measure different factors of uncertainty in clinical decision making from the perspectives of Iranian clinical physicians (CUMQ) which is not seen together in the previous measurement tools. So, this study closes the scientific gap for the lack of comprehensive assessment tools to measure clinical uncertainty factors that have reliability and validity for the Iranian medical community.

## Methods

### Study design

This questionnaire has been derived from a mixed-method study since March 2019 to January 2021 at Iran University of Medical Sciences.

### First phase: a systematic review

At first, the literature was reviewed based on to derive raw items [5, 6, 12, 18, 19]. A systematized review of research papers relating to the reliability and validity of uncertainty in clinical decision-making questionnaires and its dimensions was conducted throughout 2019 to 2020. We searched Medline/PubMed, Web of Science, Scopus, and databases by combining three terms: 1) uncertainty, 2) “clinical decisions” OR “clinical decision-making”, 3) physician OR specialist, and 4) measure OR measurement OR questionnaire.

The initial search and abstract review were conducted by SG in 2019, with articles retrieved whether they were considered to be relevant on the basis of the abstract or the abstract did not include sufficient information on which a judgement could be based. SG then screened full-text articles. A second search, in 2020 was performed in order to update the review and was supplemented with additional articles.

#### Inclusion and exclusion criteria

The inclusion criteria were; a) relating to clinical uncertainty, d) assessing uncertainty factors in clinical decisions, e) providing a measurement for uncertainty in clinical decision-making, and f) reliability and/or validity were assessed for the measurement. Articles which were not written in English-language and published papers prior to 2008 were excluded.

#### Data extraction of literature review

**Fig. 1:**
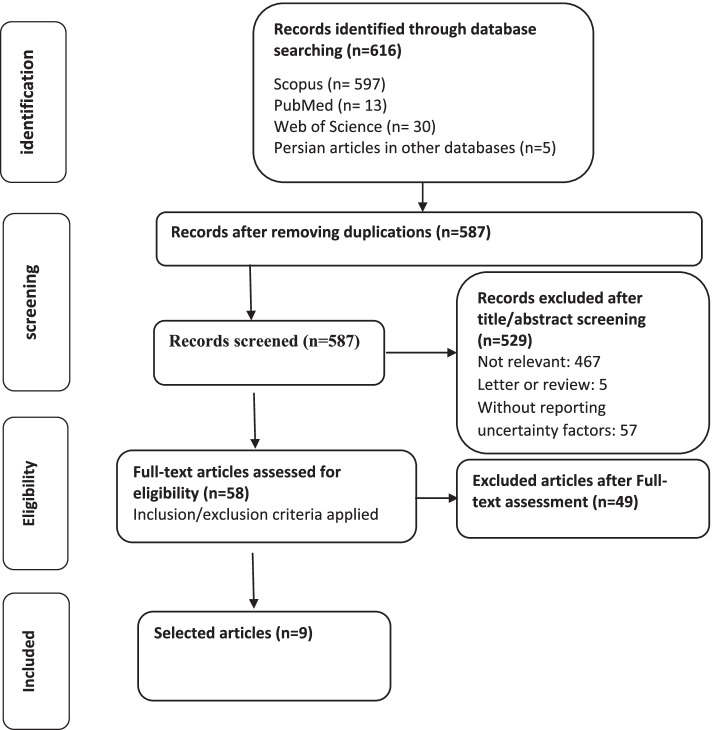
PRISMA flow diagram of the systematic review process

As described in Fig. 1, a total of 616 records identified of which 587 articles remained after removing duplicated references.

We extracted information from each paper into a spreadsheet. Data collected included: a) Bibliographic information, b) A description of the Questionnaires, c) Classification of the Questionnaires (Educational or Clinical Uncertainty), d) Sample (e.g. medical students, residents & physicians), e) Validity assessments, and f) Reliability and internal consistency estimates.

At last, 9 articles met inclusion criteria and remained in the analysis. Table 1 shows the details of included studies.

**Table 1:** Main characteristics of the articles included in the reviewphase

Code	Title	Author	Year	Derived items to help finding uncertainty determinants
1	Twelve tips for thriving in the face of clinical uncertainty	Gheihman et al. [20]	2020	-Everyone should know their reaction in cases of uncertainty - One must identify the type and source of uncertainty -Identify cognitive biases - Planning for uncertainty: Use a secure network and track - Do not worry about loneliness, rely on your colleagues - Culture building: a pattern of accepting the inherent uncertainty of clinical medicine -Promote curiosity among students more than certainty -Be clear about the level of uncertainty -Include uncertainty explicitly in medical education courses -Talk to patients about uncertainty in public -Use patients as allies in joint decision making
2	Decision-making under uncertainty in environmental health policy: newapproaches	Reis and Spencer [21]	2019	- Accepting the uncertainty of science - Teaching the principle of scientific uncertainty to all stakeholders and policy makers
3	Decision-Making under Uncertain Conditions: The Internist, as a Director of the Diagnostic/Therapeutic Pathway in Grey Zones	Tirotta and Durante [18]	2018	-Predict frequently asked questions about the patient's problem (problem, intervention, exposure, outcome) - Determine the best available evidence and make a decision - Perform critical evaluation and information transfer - Patient and specialist consultation - Another doctor's opinion -Referral to another medical center with a higher number of similar cases admitted to specific cases -Consider a collection of patients, physicians, evidence, Gray Literature, socioeconomic context, and expertise in related fields
4	Uncertainty of Physicians and Patients inMedical DecisionMaking	Dhawale et al. [22]	2017	-Accepting and understanding uncertainty -Effective communication between doctor and patient - Predict the risk of disease consequences based on its recurrence in the past
5	Uncertainty and objectivity in clinical decision making: a clinicalcase in emergency medicine	Engebretsen et al. [23]	2016	- Analysis of emergency cases - Active questions
6	The Diagnosis, Prognosis, and Treatment of Medical Uncertainty	Wray and Loo [12]	2015	- Teaching students the inherent medical uncertainty - Supervising schools to change the prevailing view of uncertainty - Share decisions with the patient - Using experienced doctors and consulting with them - Refer to evidence-based sources - Refer to the guidelines Opportunity to write with feedback and ways such as role modeling to express uncertainty
7	Recognizing and Responding to Uncertainty: A Grounded Theory Uncertainty	Cranley et al. [24]	2012	- Imagine the situation for yourself - Consult with other colleagues - Search for evidence and sources - Training programs to increase the ability to deal with uncertainty
8	Risk, Uncertainty and Indeterminacy in Clinical Decisions	Strand et al. [25]	2010	Risk, consequences and effectiveness of different treatments - Severe uncertainty about the patient's condition and ignorance of his characteristics - Unawareness of the consequences of the decision and the side effects of the treatment of choice - Uncertainty of clinical problem
9	Resident uncertainty in clinical decision making and impact on patient care: a qualitative study	Farnan et al. [13]	2008	-Consult with people around you - Consult with experienced doctors

### Second phase: a qualitative study

Through the second step, 30 participants were selected by purposive sampling. Data were collected using semistructured interviews, which continued until data saturation. Data were analyzed according to conventional content analysis approach. Transcribed interviews were considered as analysis units. Meaning units were extracted from interview texts, after several times of reading and understanding sentences. Codes were formed that may be referred to as labels for meaning units. Next, categories were created from grouping of similar codes. Theme is the foundation for contents in categories.

The residents, specialists and sub-specialist of four main clinical groups, including obstetrics, surgery, pediatrics and internal medicine of Iran University of Medical Sciences were selected using purposive sampling method. Therefore, other practicing physicians were excluded. consent form was secured from the study participants. Data gathering was conducted after fulfilling the written consent in accordance with the Declaration of Helsinki from participants. The informed consent let the participants be aware of all options. They could withdraw from the study at any time. All interviews were recorded using a digital recorder after obtaining all participants’ permissions in a quite room and at the most suitable hour for the participants.

As mentiond, We implemented in-depth interviews with clinical physicians to get a more comprehensive view of uncertainty determinants. In this part, we asked the doctor to express his experience of clinical uncertainty. In addition, during the interview, based on the interview process, we asked them to explain anything that caused uncertainty.

The recorded interviews were transcribed verbatim (SG) and analyzed through conventional content analysis method. At first, the transcript of each interview was read several times, then, related meaning units were identified. In the next step, each condensed meaning unit was given a code and codes with similar concept were formed sub-categories. After that, similar sub-categories grouped as categories. The last step of the analysis was forming main categories which were done according to interpretations of meanings in the sub-categories.

The participants received codes and categories whom were asked to check the appropriateness and correctness of the extracted results in order to member-check. According to the result of the qualitative phase, determinants of uncertainty in clinical decision-making consisted of three main categories; individual determinants (can be related to the physician or patient), dynamics of medical sciences, and diagnostic and instrumental constraint (lack of efficient diagnostic tests and unknown etiology).

### Participants and sampling

**Fig. 2:**
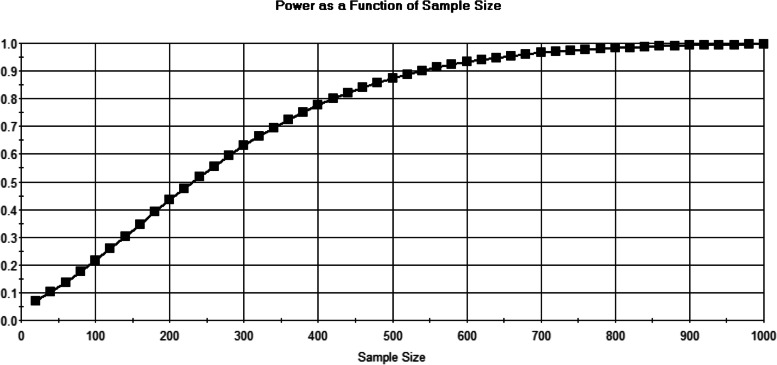
Sample size estimation via sample power software

Sampling was done in two stages; 1) sampling for content validity, 2) sampling for testing the model. The simple random sampling method was used for content validity of the items. According to the statistics of professors and assistants of the four major clinical groups of Surgery, Internal Medicine, Gyncology and Pediatrics the sample of 30 people with an error of 0. 5%, and the confidence of 99%, the test power of 80% and effect size of 0. 03 was selected using Sample Power Sampling Software (SPSS). The sample included 12 assistant professors, 6 associate professors, and 12 full professors (Fig. 2).

According to the references, ascertaining the sample size for the validity assessing phase has always been partly unpredictable. In order to have a sufficient control over the agreement, at least five people are recommended and the maximum number of judges has not been determined yet [26].

And to test the model, the statistical population consisted of faculty members and assistants of the four major clinical groups of Surgery, Internal Medicine, Gyncology and Pediatrics. As demonstrated in Fig. 2, the total sample size was estimated 431 individuals by SPSS based on target population. Finally, 391 CUMQ were completed and entered into the analysis. The sample size was chosen through stratified sampling method.

### Data analysis

The CUMQ was finally completed by 391 clinical physicians and residents of four major clinical groups after eliminating not completed questionnaires to evaluate its validity and reliability. To analyze the data, we used Smart PLS and SPSS softwares. SmartPLS is one of the prominent software applications for Partial Least Squares Structural Equation Modeling (PLS-SEM). Smart PLS presents path modeling estimations the partial least squares (PLS) approach, as the second generation of structural equation methods, has opened new horizons for researchers. The reason for choosing this approach is that unlike the covariance-based approach, it has less dependence on the sample size, level of measurement of variables and the normality of the distributed data. It can be said that the partial least squares (PLS) approach requires fewer conditions than similar techniques to structural equations such as LISREL and AMOS. PLS is more suitable for real applications especially when the models are more complex, it will be more desirable to use this approach. Of course, the main advantage of the partial least squares (PLS) approach is that it requires fewer samples than other approaches such as LISREL and AMOS. In fact, PLS has no sample size limit [27].

In this research, using the partial least squares approach, measurement models through validity and reliability analysis and first and second order confirmatory factor analysis will be studied. In general, the test criteria of the measurement model in the partial least squares (PLS) approach are as follows.

To assess the structural model, the corresponding t-values through a bootstrapping procedure, is suggested based on available evidence [28]. In bootstrapping technique, T-statistics generate for significance testing of both the inner and outer models. For this purpose, large number of sub samples are taken from the original sample with replacement to give bootstrap standard errors, which gives approximate T-values for significance testing of the structural path [27].

Accordingly, the values of two indicators’ quality of the model adjustment should be evaluated: relevance or predictive validity (Q^2^) and effect size (f^2^) [29]. The Q^2^ evaluates how much the model approaches were expected. For these criteria, values greater than zero should be obtained. The Q^2^ determine useful each construct is for the adjustment model. Values of 0. 02, 0.15 and 0. 35 are considered small, medium, and large, respectively [30].

To assess the content validity of CUMQ, content validity index (CVI) and content validity ratio (CVR) have been used. To obtain CVI, the panel of 10 experts was held to rate each item in terms of its relevance to the studied subject. The 4-point scale was used to avoid a neutral opinion and the item rating were 1 = not relevant, 2 = somewhat relevant, 3 = quite relevant, 4 = highly relevant [31, 32]. The minimum acceptable value for the CVI is literallyassumed0. 79, and if the CVI item is less than 0. 79, that item will be removed.

According to the Lawshe test, CVR is computed to specify whether an item is necessary for operating a construct in a set of items or not. For this, the expert panel was asked to consider score 1for essential items, 2 for useful but not essential items, and 3 for not necessary ones. The formula for computation of CVR = (Ne – N / 2) / (N / 2); Ne is the number of panelists representing “essential” and N is the total number of participants. The numeric value of CVR ranges from -1 to 1 [33]. Responses were calculated based on the CVR formula and matched to the Lawshe table. Numbers above 0. 62 were accepted.

Since Cronbach’s alpha is a traditional method to measure internal consistency reliability, and “composite reliability” has been suggested as a replacement for it [27]; In this study, we used both methodsto assess the reliability of CUMQ. To measure the construct validity of CUMQ, the convergent and discriminant validity which are measures of construct validity were evaluated [27]. In sequence, the initial aspectsof the measuring model to be observed are the convergent validities which are obtained by observations of the Average Variance Extracted (AVEs) for each latent variable (LV). To have a convergent result, the AVEs should be greater than 0. 50 [29].

To evaluate the discriminant validity of the model, the criteria of Fornell and Larcker was applied. This criteria compares the square roots of the AVE values of each construct with the Pearson correlations between the constructs (or LV) [29]. Moreover, the factor structure of CUMQwas confirmed using confirmatory factor analysis.

## Results

In this study, 391 physicians completed the questionnaire. A total of 245 (62. 7%) included male participants and 146 (37. 3%) of female ones. Frequency distribution of respondents by specialty indicates that 131 (33. 5%) were surgeons, 105 (26. 8%) were internal medicine specialists, 100 (25. 6%) were gynecologists and 55(14. 1%) were pediatricians (Table 2).

**Table 2:** Socio-demographic characteristics of the studied sample

Variable	n (percentage)
**Age (Mean ± SD)**	43.21 (± 8.38)
Work experience (Mean ± SD*)	12.29 (8.03)
**Gender**	
Male	245 (62.7)
Female	146 (37.3)
**Level of education**	
Resident	138 (35.3)
Specialty	80 (20.4)
Fellowship	109 (27.9)
Sub-specialty	64 (16.4)
**Major**	
Surgery	131 (33.5)
Internal medicine	105 (26.8)
Pediatrics	55 (14.1)
Gynecology	100 (25.6)
**Job rank**	
Assistant	156 (39.9)
Fellowship assistant	95 (24.3)
Faculty member	140 (35.8)

### Content validity

The CVR and CVI ranged from 0. 80 to 1. 00 for CUMQ items (Table 3). Based on the results, all items had an acceptable coefficient and remained in the questionnaire at this stage. Preliminary version of CUMQ showed high content validity.

**Table 3:** CVI & CVR statistics

Items	CVR	CVI
Q1	1.00	1.00
Q2	0.80	0.90
Q3	1.00	1.00
Q4	0.80	1.00
Q5	1.00	1.00
Q6	0.80	0.90
Q7	1.00	1.00
Q8	1.00	1.00
Q9	1.00	1.00
Q10	1.00	1.00
Q11	0.80	1.00
Q12	1.00	1.00
Q13	1.00	1.00
Q14	1.00	1.00
Q15	1.00	1.00
Q16	1.00	1.00
Q17	0.80	0.80
Q18	1.00	1.00
Q19	0.80	0.90
Q20	1.00	1.00
Q21	1.00	1.00
Q22	1.00	1.00
Q23	0.80	0.90
Q24	1.00	1.00
Q25	0.80	0.90
Q26	0.80	0.90
Q27	1.00	1.00
Q28	1.00	1.00
Q29	0.80	0.90
Q30	1.00	1.00
Q31	0.80	0.90

### Confirmatory factor analysis model

According to the extracted model, factor loading and t-value for each indicator of uncertainty is reported. Five dimensions of clinical uncertainty included general determinants, individual determinants of the physician, individual determinants of patient, dynamics of medical sciences, diagnostic and instrumental limitations were confirmed. As demonstrated in Table 4, 24 questions which had an appropriate factor load on their latent variable, entered into the model (7 questions with factor loading less than 0.6 were omitted). These factor loads are significant with respect to the t-value at significance level of 0. 01. Therefore, these items had a necessary accuracy to measure their respective structures and were entered to the final analysis.

**Table 4:** Result of factor analysis containing standardized determinants of clinical uncertainty

Variable	Latent variable	Indicators	Factor loading	*t*-value	*p*-value
**Uncertainty**	General determinants	Q1	0. 744	24. 389	0. 001
		Q2	0. 692	20. 359	0. 001
		Q3	0. 808	36. 739	0. 001
		Q4	0. 821	44. 187	0. 001
		Q5	0. 781	32. 471	0. 001
	Individual determinants of the physician	Q6	0. 796	30. 407	0. 001
		Q7	0. 833	45. 646	0. 001
		Q8	0. 850	53. 895	0. 001
		Q9	0. 632	16. 322	0. 001
	Individual determinants of the patient	Q12	0. 663	19. 765	0. 001
		Q13	0. 847	44. 034	0. 001
		Q14	0. 754	25. 958	0. 001
		Q15	0. 796	23. 844	0. 001
	Dynamics of medical science	Q16	0. 710	25. 949	0. 001
		Q17	0. 735	24. 909	0. 001
		Q18	0. 783	36. 052	0. 001
		Q19	0. 775	36. 356	0. 001
		Q20	0. 651	18. 153	0. 001
		Q22	0. 649	17. 173	0. 001
	Diagnostic and instrumental limitations	Q23	0. 696	22. 802	0. 001
		Q25	0. 655	15. 987	0. 001
		Q28	0. 769	29. 066	0. 001
		Q29	0. 739	22. 408	0. 001
		Q30	0. 653	15. 471	0. 001

**Fig. 3:**
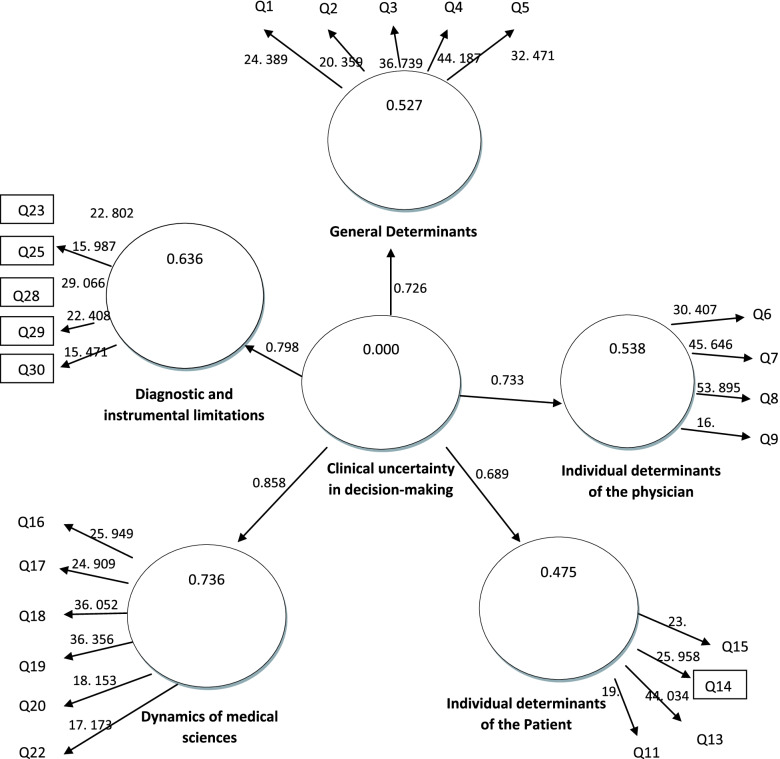
Bootstrapping results

As Fig. 3 shows the output of Smart-Pls software regarding the measurement correction model related to research variables was assessed.

### Reliability

The value of composite reliability and Cronbach’s alpha for all the five LVs of uncertainty was above the threshold value of 0. 7. Therefore, the reliability of CUMQ is confirmed. The results of the composite reliability and Cronbach’s alpha describe that the research instrument is a reliable survey tool to measure five clinical uncertainty determinants’ domains, respectively (Table 5).

**Table 5:** The internal consistency reliability of CUMQ based on dimensions of uncertainty

Variable	Composite reliability	Cronbach’s alpha	AVE
General determinants	0. 879	0. 829	0. 593
Individual determinants of the physician	0. 862	0. 783	0. 612
Individual determinants of the patient	0. 845	0. 757	0. 579
Dynamics of medical science	0. 865	0. 812	0. 517
Diagnostic and instrumental limitations	0. 830	0. 744	0. 500

### Validity

In order to measure the validity of CUMQ, convergent and discriminant validities were applied:

#### Convergent validity

To check convergent validity, each latent variable’s AVE is evaluated. As Table 5 shows, all of the AVE values are greater than the acceptable threshold of 0. 5 [29]. So, convergent validity of CUMQ is confirmed.

#### Discriminant validity

To evaluate the discriminant validity, the criteria of Fornell andLarcker and cross loading wereassessed. Table 5 illustrates the results of the Fornell-Larcker criterion assessment with the reflective construct of the latent variable general determinant AVE is found to be 0. 593 (Table 5) hence its square root becomes 0. 777. This number is larger than the correlation values in the column of general determinant. As for the reflective construct of “individual determinants of the physician”, it has a value of 0. 782 for the square root of its AVE which is greater than “individual determinants of patient”(0. 439), “dynamics of medical sciences”(0.507) and “diagnostic and instrumental limitations” (0. 501). Similar observationswere also conducted for the other LVs. Therefore, the result of Fornell-Larcker indicates that discriminant validity is well established (Table 6).

**Table 6:** Fornell-Larcker criterion for the five uncertainty dimensions construct

Indicator	General determinants	Individual determinants of the physician	Individual determinants of patient	Dynamics of medicalscience	Diagnostic and instrumental limitations
General determinants	0. 777				
Individual determinants of the physician	0. 483	0. 782			
Individual determinants of patient	0. 364	0. 439	0. 760		
Dynamics of medical science	0. 506	0. 507	0. 560	0. 719	
Diagnostic and instrumental limitations	0. 467	0. 501	0. 423	0. 620	0. 707

Another procedure which was applied to identify discriminating validity of the model, names cross loading. Table 7 shows the cross-loadings for each indicator which reflected on 5 different latent constructs (i. e. general determinants, etc.). Items Q1 to Q5 load high on its corresponding construct (general determinant) and lower on other four remained constructs (Table 7).

**Table 7:** Cross loadings for the constructs of the dimensions of clinical uncertainty

Items	General determinants	Individual determinants of the physician	Individual determinants of patient	Dynamics of medical science	Diagnostic and instrumental limitations
Q1	0. 745	0. 306	0. 215	0. 279	0. 247
Q2	0. 692	0. 325	0. 214	0. 342	0. 256
Q3	0. 808	0. 389	0. 353	0. 410	0. 378
Q4	0. 821	0. 396	0. 328	0. 453	0. 444
Q5	0. 781	0. 431	0. 270	0. 436	0. 430
Q6	0. 402	0. 796	0. 343	0. 420	0. 374
Q7	0. 359	0. 833	0. 380	0. 340	0. 367
Q8	0. 372	0. 850	0. 368	0. 450	0. 471
Q9	0. 380	0. 631	0. 279	0. 366	0. 342
Q12	0. 423	0. 486	0. 663	0. 482	0. 401
Q13	0. 244	0. 331	0. 847	0. 434	0. 292
Q14	0. 231	0. 242	0. 753	0. 389	0. 314
Q15	0. 141	0. 203	0. 769	0. 354	0. 239
Q16	0. 372	0. 383	0. 563	0. 710	0. 405
Q17	0. 315	0. 374	0. 390	0. 735	0. 460
Q18	0. 391	0. 390	0. 387	0. 783	0. 423
Q19	0. 386	0. 349	0. 404	0. 775	0. 456
Q20	0. 325	0. 286	0. 285	0. 651	0. 408
Q22	0. 389	0. 394	0. 367	0. 649	0. 522
Q23	0. 423	0. 382	0. 313	0. 526	0. 696
Q25	0. 301	0. 423	0. 384	0. 409	0. 655
Q28	0. 356	0. 271	0. 258	0. 451	0. 769
Q29	0. 264	0. 290	0. 208	0. 345	0. 737
Q30	0. 273	0. 380	0. 310	0. 419	0. 653

The Q^2^ for all the five determinants of clinical uncertainty was greater than 0.15 (Q^2^ for general determinants = 0. 308, individual determinants of the physician = 0. 321, individual determinants of the patient = 0. 258, dynamics of medical sciences = 0. 373, and for diagnostic and instrumental limitations = 0. 300).

## Discussion

As there is an essential need to evaluate the clinical uncertainty among physicians in order to reduce that towards better care, this study explored the main requirements to introduce a comprehensive assessment tool to measure determining dimensions of clinical uncertainty that have reliability and validity for the Iranian medical community. The CUMQ (Additional file 1) is designed to measure determinants of uncertainty in clinical decision making from the clinical specialists, subspecialists and residents’ points of view which provides a unique self-report tool to identify and measure various dimensions of clinical uncertainty reasons. For this purpose, a mixed-method study including a systematic review and a qualitative study were implemented. The Iranian CUMQ questionnaire was assessed by using the PLS-SEM approach.

Through the qualitative phase of study, it has been demonstrated that uncertainty in clinical medicine is impossible which can lead to the inability to identify and determine the meanings of disease-related events or the inability to definitively predict disease events. In order to assess the general uncertainty, which can not be eliminated but reduced, various aspects of uncertainty in CUMQ are considered to be assessed. This is important because it can summarize the general state of uncertainty in the clinical context.

Despite the nature of medical uncertainty, which is part of dynamic medical science, it seems that evaluating and accepting uncertainty in clinical decision-making, which is measureable using CUMQ can improve residents and physicians’ comfort, create agent coaching, create a culture of respect throughout the hospital, and strengthens the right doctor-patient relationship and patient care. Enhanced awareness of uncertainty understanding can be important factors in improving work environment and the quality of care given to patients [34]; So the CUMQ will be helpful for residents and physicians to assess the level of uncertainty to prepare themselves in order to encounter uncertain conditions in practice.

Although some questionnaires had been developed in previous literature in which physicians’ reactions towards uncertainty have been focused on [14, 16, 17], there is still scarce research with an objective of validating instruments that measure various dimensions and determinants among clinical physicians worldwide.

The dimensions being measured by CUMQ would be addressed as general determinants, individual determinants associated with the physician, individual determinants associated with the patient, dynamics of medical sciences, and diagnostic and instrumental limitations.

The CUMQ has showed a high content validity with the CVR and CVI range between 0. 80 and 1. 00. Confirmatory factor analysis (CFA) results have also shown a good fit with salient loadings higher than 0.50. The initial 31 items were subjected to enter into factor analysis model. Twenty-four items were remained in CUMQ due to their high factor loadings.

The reliability analysis for CUMQ reported Cronbach’s alpha value of 0. 829 for general determinants, 0. 812 for dynamics of medical sciences, 0. 783, 0. 757, and 0. 744 for individual determinants of the physician, individual determinants of the patient, and diagnostic and instrumental limitations, respectively. The composite reliability was also above the value of 0. 7 for all the five dimensions. Therefore, the reliability of CUMQ was confirmed. Due to lack of a similar study which validated the exact dimensions of clinical uncertainty, we inevitably compared the findings with studies with albeit small similarities. The reliability of ‘Physician Reaction to Uncertainty’ (PRU) Questionnaire is investigated in different versions and all of them reported acceptable internal consistency of Cronbach’s alpha values of more than 0. 70 for all scales [14, 17].

The AVE value for all dimensions of CUMQ was above 0.5 which demonstrated that the model has established distinct levels of convergent validity [29]. For the discriminant validity, the results of cross loading and Fornell-Larker criterion showed that discriminant validity is well established with high factor loading. After that, we evaluated the quality of the model adjustment through predictive validity (Q^2^) and effect size (f^2^). The Q^2^ for all the five determinants of clinical uncertainty was considered greater than 0.15 which shows acceptable quality of the measurement model [30].

## Strenghts and limitations

The present study led to developing the valid and reliable questionnaire addressed CUMQ, to assess clinical uncertainty among practicing physicians and clinical residents with all determing aspects for the first time.

To mention the limitations, it can be referred to PLS_SEM that has a limitation. It has to identify the reliability and validity with less statistical methods compared to covariance constructs. That is the reason to use resampling procedures e.g. bootstrapping to get information about the validity and reliability of the model. This is a disadvantage but with an increased sample size this disadvantage is not apparent**.**

## Conclusion

Based on the experiences of four major clinical groups residents and physicians and according to literature, assessing the clinical uncertainty would be considered as the first step towards enhancing definite clinical decision making. The findings of this study confirmed the validity and reliability of CUMQ in measuring determinants of clinical uncertainty among Iranian physicians and residents. The CUMQ that is developed for the first time fulfills the clinical uncertainty and how to deal with it. It is recommended that the questionnaire be used in different contexts to find its validity and reliability.

## Supplementary Information

**Additional file 1.** Clinical Uncertainty Measurement Questionnaire (CUMQ).

**Additional file 2.**

## Data Availability

The datasets generated and/or analysed during the current study are not publicly available due but are available from the corresponding author on reasonable request.

## Footnotes

1 Smart PLS.
