# Supplementary material for: Developing and validating of the Clinical Uncertainty Measurement Questionnaire (CUMQ) among practicing physicians and clinical residents in Iran
Source: BMC Med Educ. 2022 Jun 16;22:462. doi: 10.1186/s12909-022-03444-1 (PMC9202180; doi:10.1186/s12909-022-03444-1)
Supplement: Supplementary file 1 — Additional file 1. Clinical Uncertainty Measurement Questionnaire (CUMQ). [file 12909_2022_3444_MOESM1_ESM.docx]

**Clinical Uncertainty Measurement Questionnaire (CUMQ)**

Dear Colleague

This questionnaire has been compiled to conduct the research project of Shirin Ghanavati, PhD thesis, entitled "Developing and validating of Clinical Uncertainty Measurement Questionnaire among practicing physicians and clinical residents of four main clinical groups" with the aim of assessing uncertainty in clinical decision making.

Uncertainty in clinical decision making from the physicians' point of view can be defined as "the inability to accurately determine the cause or causes of the patient's main problem, which leads to multiple diagnoses and subsequent multiple decisions to treat the patient. "

Due to the fact that your answer to the questions will remain completely confidential, please answer the following questions carefully and in case of any questions, please email [shiringhanavati@ymail. com](mailto:shiringhanavati@ymail.com).

Age: ……………………. .

Gender: Male Female

Educational Status: Specialtty Fellowship Subspecialty

Major Group: Surgery Internal Medicine Pediatrics GYN

How long have you been working as a general physicain?. . . . . . . . . . . . . . . . . . . . . . . . . . . . . . . . . . . . . . . . . . . . . . . . . . . . . . . .

How long have you been working as a specialist?. . . . . . . . . . . . . . . . . . . . . . . . . . . . . . . . . . . . . . . . . . . . . . . . . . . . . . . . . . . . . . . . . . . . . .

Have you ever participated in “Uncertainty in Clinical Decision Making” courses?. . . . . . . . . . . . . . . . .

To what extent have you encountered the following in clinical decision making?

| No. | The extent of the impact of each of the determinants of uncertainty | Very much | Much | Up to certain level | Low | Very low | |
| --- | --- | --- | --- | --- | --- | --- | --- |
| **Uncertainty Caused by General Determinants** | | | | | | | |
| Q1 | I have encountered uncertainty in the clinical decision for the patient. |  |  |  |  |  | |
| Q2 | In my field, uncertainty in decision making is common. |  |  |  |  |  | |
| Q3 | In dealing with patients with the underlying disease, I have not encountered clinical uncertainty. |  |  |  |  |  | |
| Q4 | In the face of the patient, I experience uncertainty in clinical decisions due to existential uncertainty (uncertainty about the future). |  |  |  |  |  | |
| Q5 | The cost-benefit of each treatment has led to uncertainty in my clinical decision about the disease. |  |  |  |  |  | |
| **Uncertainty Caused by Individual Determinants of the Physician** | | | | | | | |
| Q6 | I have sometimes encountered a lack of consensus with other colleagues on diagnosis. |  |  |  |  | |  |
| Q7 | I have encountered uncertainty in clinical diagnosis due to simultaneous involvement of several patient organs. |  |  |  |  | |  |
| Q8 | The existence of multiple treatments for a disease has led to uncertainty in my clinical decision about the patient. |  |  |  |  | |  |
| Q9 | I have encountered clinical decision uncertainty in life-threatening diseases. |  |  |  |  | |  |
| **Uncertainty Caused by Individual Determinants of Patient** | | | | | | | |
| Q10 | Presenting a history with the patient's companion causes uncertainty in the clinical diagnosis. |  |  |  |  | |  |
| Q11 | Different patient dialects and literature cause uncertainty in clinical decision making and diagnosis. |  |  |  |  | |  |
| Q12 | The patient's level of awareness of his/her health status helps the physician make a definitive diagnosis. |  |  |  |  | |  |
| Q13 | The patient's mental state has no effect on the physician's definitive diagnosis. |  |  |  |  | |  |
| **Uncertainty Caused by Dynamics of Medical Science** | | | | | | | |
| Q14 | In my field, the unpredictability of the patient's condition leads to uncertainty. |  |  |  |  | |  |
| Q15 | Insufficient uncertainty about the progression of the disease is one of the reasons for the uncertainty in my decision. |  |  |  |  | |  |
| Q16 | I am facing uncertainty due to the constant change in medical science in my field. |  |  |  |  | |  |
| Q17 | I am facing uncertainty due to emerging and rare diseases. |  |  |  |  | |  |
| Q18 | The rapid advancement of medical science has led to certainty in diagnosis and clinical decision making. |  |  |  |  | |  |
| Q19 | Things like telemedicine cause uncertainty in my diagnosis and clinical decision. |  |  |  |  | |  |
| Q20 | I have encountered uncertainties due to the limitations of using diagnostic tests. |  |  |  |  | |  |
| Q21 | The low accuracy of diagnostic measurement tools is one of the causes of decision uncertainty that I have encountered in my field. |  |  |  |  | |  |
| Q22 | The limitation of performing diagnostic tests due to the patient's financial shortcomings has led to uncertainty in my diagnosis and clinical decision. |  |  |  |  | |  |
| Q23 | The lack of general treatment guidelines in the guidelines and the asymmetry of diagnostic resources are among the factors of uncertainty in my decision. |  |  |  |  | |  |
| Q24 | The fact that the symptoms of the disease are not necessarily visible at the first visit makes me uncertain. |  |  |  |  | |  |
