# Supplementary material for: Developing and validating of the Clinical Uncertainty Measurement Questionnaire (CUMQ) among practicing physicians and clinical residents in Iran
Source: BMC Med Educ. 2022 Jun 16;22:462. doi: 10.1186/s12909-022-03444-1 (PMC9202180; doi:10.1186/s12909-022-03444-1)
Supplement: Supplementary file 2 — Additional file 2. [file 12909_2022_3444_MOESM2_ESM.pdf]

Search Strategy:

((TS=(uncertainty)AND (TS=("clinical decision" OR "clinical decision-making") AND (TS=(physicain OR specialist) AND (TS=(measure OR measurement OR questionnare))) AND LANGUAGE: (English) AND document types: (Article)

Indexs-SCI-EXPANDED, SSCI Timespan=2008-2020
